# Zinc Ion-Based Switch-on Fluorescence-Sensing Probes for the Detection of Tetracycline

**DOI:** 10.3390/molecules27238403

**Published:** 2022-12-01

**Authors:** Yan-Cen Zhan, Jia-Jen Tsai, Yu-Chie Chen

**Affiliations:** 1 Department of Applied Chemistry, National Yang Ming Chiao Tung University, Hsinchu 300, Taiwan; 2 International College of Semiconductor Technology, National Yang Ming Chiao Tung University, Hsinchu 300, Taiwan

**Keywords:** tetracycline, zinc ion, fluorescence-based sensing, aggregation-induced emission

## Abstract

Tetracycline (TC) is an antibiotic that has been widely used in the animal husbandry. Thus, TC residues may be found in animal products. Developing simple and sensitive methods for rapid screening of TC in complex samples is of great importance. Herein, we demonstrate a fluorescence-sensing method using Zn^2+^ as sensing probes for the detection of TC. Although TC can emit fluorescence under the excitation of ultraviolet light, its fluorescence is weak because of dynamic intramolecular rotations, leading to the dissipation of excitation energy. With the addition of Zn^2+^ prepared in tris(hydroxymethyl)amino-methane (Tris), TC can coordinate with Zn^2+^ in the Zn^2+^-Tris conjugates to form Tris-Zn^2+^-TC complexes. Therefore, the intramolecular motions of TC are restricted to reduce nonradiative decay, resulting in the enhancement of TC fluorescence. Aggregation-induced emission effects also play a role in the enhancement of TC fluorescence. Our results show that the linear dynamic range for the detection of TC is 15–300 nM. Moreover, the limit of detection was ~7 nM. The feasibility of using the developed method for determination of the concentration of TC in a complex chicken broth sample is also demonstrated in this work.

## 1. Introduction

Tetracycline (TC) is a common antibiotic that can effectively inhibit the growth of pathogens, including Gram-positive and Gram-negative bacteria [1]. It has been used to treat bacterial infections and as feed additives to promote the growth of livestock [2]. However, the overuse of TC has caused some problems. For example, TC residuals have been found in animal products, which cause environmental contamination and the emergence of antibacterial strains. The appearance of TC-resistant bacterial strains is also a significant threat to human health [3]. The maximum residue level of TC in milk is 100 μg kg^−1^ (~0.2 μM), as recommended by the Food and Agricultural Organization of United Nations, the World Health Organization, and the European Union [4]. Thus, developing simple and effective methods for the sensitive and selective detection of TC is important.

A variety of methods, including high-performance liquid chromatography [5], electrochemistry [6], chemiluminescence [7], immunoassays [8], colorimetric methods [9], mass spectrometry [10], and fluorometry [11,12,13,14,15], have been used in the detection of TC. Fluorescence-based detection is considered as an effective way to detect TC because of its simplicity, high sensitivity, and fast response. To date, several fluorescence-based sensing probes such as quantum dots [16,17,18,19], metal–organic frameworks (MOFs) [20,21,22], nanoparticles [23,24], and nanoclusters [25,26] have been explored for the detection of TC. The abovementioned materials as fluorescence-sensing probes can be used to sensitively detect TC. However, the synthesis of such fluorescence probes involves multiple time-consuming synthesis steps [17,20,23]. Thus, a simple method for the generation of fluorescence probes against TC is still anticipated.

Based on the theory of hard and soft acids and bases [27], TC is a hard base because its chemical structure is rich in N- and O-containing functional groups. Thus, hard acids such as europium ions [28,29] and borderline acids such as Zn^2+^-based probes [30,31] have been synthesized and used to detect TC through fluorescence enhancement. For example, Zn^2+^ can strongly coordinate with nitrogen and oxygen-containing functional groups in TC to form Zn^2+^-TC coordination complexes [32,33,34]. The formation of a large Zn^2+^-TC conjugate network suppresses nonradiative decays and results in aggregation-induced emission (AIE) [35,36,37,38]. That is, the fluorescence of the Zn^2+^-TC conjugate is brighter than TC alone [32,33,34]. Previous studies [31,39,40] have demonstrated the feasibility of using Zn^2+^-containing MOFs as fluorescence-sensing probes against TC, and the LOD is ~17 nM [31]. Nevertheless, the preparation of MOFs is time-consuming [31]. Tris(hydroxymethyl)amino-methane (Tris) is usually used to prepare buffer. Zn^2+^ can chelate with N- and O-containing functional groups on Tris [41]. Thus, in this study, we prepared Zn^2+^ in Tris buffer as a probe against TC. AIE resulting from Tris-Zn^2+^-TC conjugates was observed. Accordingly, a new and facile fluorescence-based sensing method against TC was demonstrated.

## 2. Experimental Section

### 2.1. Reagents and Materials

L-alanine, L-histidine, L-proline, L-serine, lysine, TC, and zinc chloride were purchased from Sigma-Aldrich (St. Louis, MO, USA). Iron (III) chloride hexahydrate, potassium chloride, and sodium hydroxide were purchased from Fluka (St. Gallen, Switzerland). Hydrochloric acid (36.5−38%), magnesium sulfate, Tris, and Tris hydrochloride were obtained from J. T. Baker (Phillipsburg, NJ, USA). Sodium chloride was obtained from Duksan (Ansan, South Korea). D-glucose was purchased from Riedel-de Haën (St. Gallen, Switzerland). Chicken broth cubes were purchased from a local shop. Millex^®^GS filters (pore size: ~0.22 μm) were purchased from Millipore (Carrigtwohill, Ireland).

### 2.2. Instrumentation

Fluorescence spectra were obtained using a Horiba Jobin Yvon Fluoremax 3 spectrophotometer (Edison, NL, USA). Ultraviolet-visible (UV-Vis) absorption spectra were collected using a Varian Cary 50 UV-Vis absorption spectroscope (Palo Alto, CA, USA).

### 2.3. Examination of TC Fluorescence Enhancement in the Presence of Zn^2+^

The samples (0.3 mL) containing TC with different concentrations (0.313–4 µM) were individually incubated with aqueous zinc chloride (1.49 mM) prepared in Tris (10 mM) at pH 9 for 10 min under vortex mixing. After 10 min, photographs of the samples obtained before and after centrifugation (15,000× *g* rpm, 10 min) were taken.

### 2.4. Optimization of the Experimental Factors

The optimal Zn^2+^ concentration, pH, and the incubation time for the developed method for sensing TC were investigated. The experiments were conducted at different pH conditions in Tris buffer (pH 6, 7, 8 and 9) and glycine-NaOH buffer (pH 10). That is, the sample (0.3 mL) containing TC (0.1 µM) was vortex-mixed with aqueous zinc chloride (1.49 mM) prepared in different pH conditions for 10 min followed by the investigation by fluorescence spectroscopy. The optimal pH was determined accordingly. In addition, aqueous zinc chloride with different concentrations (3.9–62.5 mM) were prepared in Tris buffer at pH 9. The sample (0.3 mL) containing TC (0.1 µM) was vortex-mixed with aqueous zinc chloride (15 μL) prepared in Tris with different concentrations for 10 min. The resulting samples were examined by fluorescence spectroscopy. Moreover, the optimal incubation time was examined. Namely, aqueous zinc chloride (31.3 mM, 15 μL) was added to the sample (0.3 mL) containing TC (0.1 µM) under vortex mixing with different times (5, 10, 20, 30, and 60 min). The final concentration of Zn^2+^ in the mixture was 1.49 mM. The resulting samples were examined by fluorescence spectroscopy. The optimal incubation time was determined accordingly.

### 2.5. Using Zn^2+^ as the Sensing Probe against TC

The stock solution containing TC (1 mM) was prepared in methanol. The samples containing TC with given concentrations were prepared by diluting the stock solution with Tris buffer (10 mM, pH 9.0). Aqueous zinc chloride was prepared in the same Tris buffer. When conducting sensing experiments against TC, the samples (0.3 mL) containing TC at different concentrations were vortex-mixed with aqueous zinc chloride (1.49 mM) prepared in Tris (10 mM, pH 9.0) for 10 min. The resultant mixtures were examined by fluorescence spectroscopy (λ_ex_ = 397 nm).

### 2.6. Examination of Interference Effects

The examination of the interference effects of using our sensing method against TC was investigated by spiking common interference species, including Na^+^, K^+^, Mg^2+^, Fe^3+^, glucose, histidine, proline, alanine, serine, and lysine to the samples. That is, aqueous zinc chloride solution (31.3 mM, 15 µL) was added to the as-prepared samples (0.3 mL) containing specific interference species with and without TC (0.1 µM). The final concentration of Zn^2+^ in the mixture was 1.49 mM. After vortex-mixing for 10 min, the samples were examined by fluorescence spectroscopy.

### 2.7. Analysis of Simulated Real Samples

Chicken broth powder (~1 mg) obtained from a chicken broth cube was dissolved in water (50 mL) followed by filtration through a Millipore filter (pore size: ~0.2 µm). The filtrate was 10-fold diluted by Tris buffer (pH 9.0), then spiked with TC (20 nM). The standard addition method was used to estimate the concentration of TC in the as-prepared chicken broth sample. That is, the standard samples containing TC (100 nM) prepared in Tris buffer (pH 9) with different volumes were added to the chicken broth sample (60 µL). Additional Tris buffer (pH 9.0) was added to the samples to make the final volume of each sample 0.3 mL. The samples were vortex-mixed with aqueous Zn^2+^ solution (1.49 mM) prepared in Tris buffer (pH 9) for 10 min. The resultant samples were examined by the fluorescence spectroscopy. The calibration curve was plotted by using the difference of the fluorescence intensity of the wavelength at 500 and 750 nm (I_500_–I_750_) in the resultant fluorescence spectra versus the concentration of the added TC. The concentration of TC in the chicken broth sample was estimated accordingly.

## 3. Results and Discussion

### 3.1. Characterization of Tris-Zn^2+^-TC Conjugates

**Figure 1:**
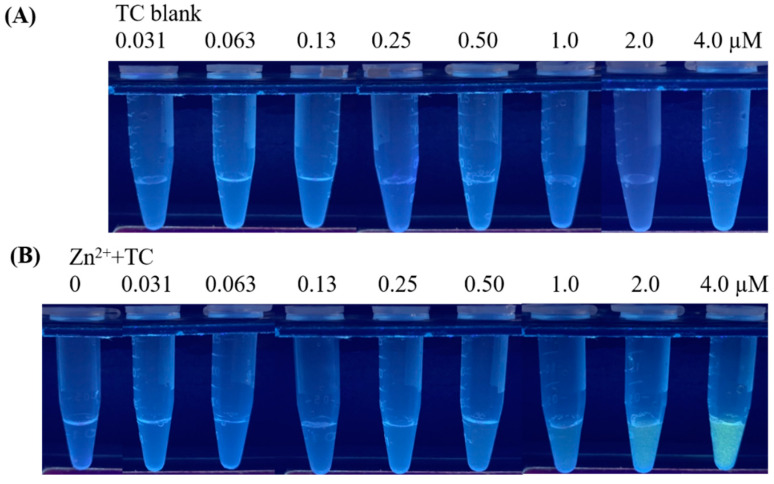
Photographs of the samples containing TC at different concentrations (**A**) without and (**B**) with adding Zn^2+^ (1.49 mM) obtained before centrifugation.

Although TC possesses fluorescence, its fluorescence was invisible when its concentration was lower than 4.0 µM under the illumination of UV light (λ_max_ = 365 nm) (Figure 1A). However, the luminescence intensity of TC was significantly enhanced with the addition of Zn^2+^ prepared in Tris because of the formation of Tris-Zn^2+^-TC conjugates (Figure 1B). Luminescence could be visualized when TC concentration was higher than 0.25 µM. The results indicated that the fluorescence of TC was greatly enhanced, with an enhancement factor up to 16, with the addition of Zn^2+^ by naked-eye detection.

**Figure 2:**
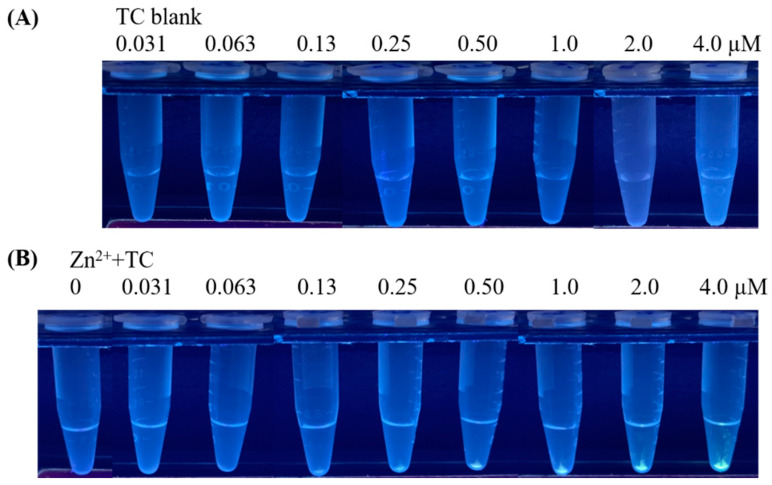
Photographs of the samples containing TC at different concentrations (**A**) without and (**B**) with adding Zn^2+^ (1.49 mM) obtained after centrifugation (15,000× *g* rpm, 10 min).

The samples shown in Figure 1 were further centrifuged to make the sensing results more visible. No precipitates were found in samples containing TC alone after centrifugation (Figure 2A). The precipitates with green-yellowish fluorescence derived from Tris-Zn^2+^-TC conjugates with TC concentration higher than 0.13 μM obtained after centrifugation were visible under the illumination of UV light (λ_max_ = 365 nm, Figure 2B). In addition, Figure S1 shows the UV-Vis absorption spectra of the samples containing TC with and without the addition of Zn^2+^ (1.49 mM) prepared in Tris. The absorption peak of the sample containing TC with the addition of Zn^2+^ was red-shifted compared with the sample containing only TC, indicating the coordination of Zn^2+^ and TC [38]. Thus, the fluorescence enhancement that resulted from the binding between TC and Zn^2+^ might be due to the AIE effect [35,38].

### 3.2. Optimization of the Experimental Parameters

**Figure 3:**
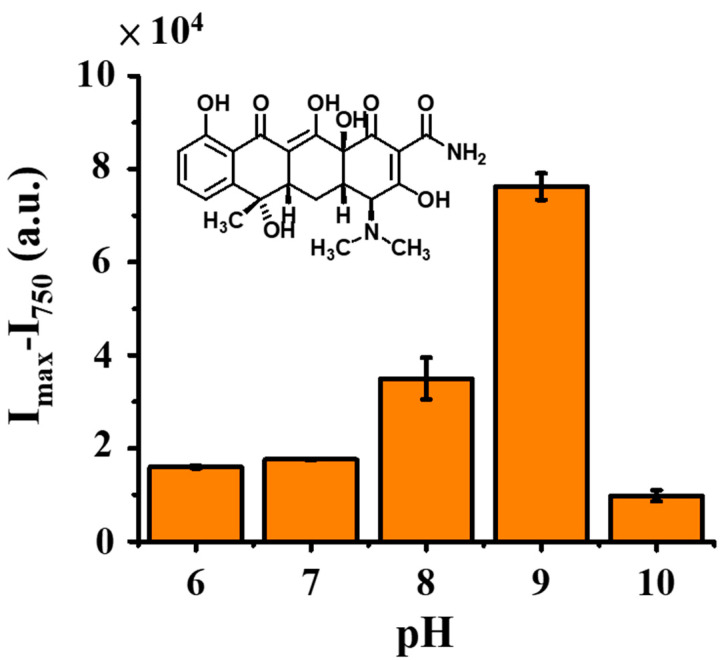
Examination of pH effects. Bar graphs showing the summarized results obtained from three replicates. Inset shows the chemical structure of TC. The sample (0.3 mL) containing TC (0.1 µM) was vortex-mixed with aqueous zinc chloride (1.49 mM) prepared in different pH conditions for 10 min, followed by the investigation by fluorescence spectroscopy.

The abovementioned results indicated the feasibility of using Zn^2+^ as a probe to detect TC. Thus, the optimal experimental parameters, including pH, Zn^2+^ concentration, and incubation time, were further examined. Supplementary Materials Figure S2 shows the representative fluorescence spectra obtained before and after adding Zn^2+^ at different pH values (6 to 10). Figure 3 shows the summarized bar graphs of the difference of the fluorescence intensity between the maximum fluorescence wavelength (I_max_) and the wavelength at 750 nm (I_750_) from three replicates. The maximum fluorescence intensity resulting from TC prepared at pH 9 was the highest. However, no fluorescence enhancement was observed at pH 10. TC possesses three pKa values (3.3, 7.7 and 9.7) [42]. As the pH value increases, TC should have an improved tendency to bind with Zn^2+^. However, Zn^2+^ has poor solubility at pH 10; thus, the fluorescence intensity of TC was not enhanced after the addition of Zn^2+^ at pH 10. Thus, pH 9 is selected as the binding pH condition in the following studies.

**Figure 4:**
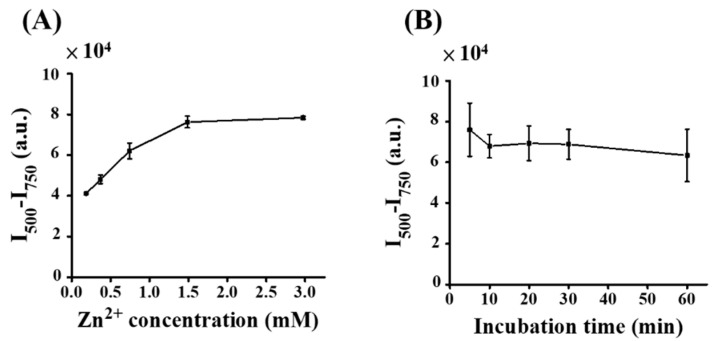
(**A**) Examination of the concentration of Zn^2+^. Graph obtained by plotting the fluorescence intensity difference at the wavelength of 500 to 750 nm (I_500_–I_750_) of the samples containing TC (0.1 µM, 300 µL) versus the concentration of Zn^2+^. Three replicates were conducted. (**B**) Examination of incubation time. Graph obtained by plotting the fluorescence intensity difference at the wavelength of 500 to 750 nm (I_500_–I_750_) of the samples containing TC (0.1 µM, 300 µL) with the addition of Zn^2+^ (1.49 mM) at pH 9 versus incubation time. Three replicates were conducted.

The optimal concentration of Zn^2+^ in the sensing of TC was further investigated. Figure S3A shows the representative fluorescence spectra of the sample containing TC (0.3 mL) added with Zn^2+^ (15 μL) at different concentrations (0–62.5 mM) prepared in Tris at pH 9. That is, the final concentration of Zn^2+^ in the sample varied from 0 to 2.98 mM. Figure 4A shows the summarized results from three replicates. The maximum fluorescence intensity at 500 nm reached the highest as the concentration of Zn^2+^ in the sample was increased to 1.49 mM, whereas the fluorescence intensity of the emission band did not change as the concentration of Zn^2+^ was further increased to 2.98 mM. The results indicated that Zn^2+^ with the concentration of 1.49 mM in the sample had the best fluorescence enhancement toward TC. Moreover, the incubation time was further examined. Figure S3B shows the representative fluorescence spectra of the samples containing TC with the addition of Zn^2+^ at different incubation times. Figure 4B shows the summarized results from three replicates. The fluorescence spectra remained unchanged after 10 min, indicating that equilibrium was achieved. The decrease in fluorescence intensity after incubation for more than 30 min was due to precipitates resulting from Tris-Zn^2+^-TC conjugates, which became evident when the incubation time was further extended. Based on the abovementioned results (Figure 3 and Figure 4), the optimal pH, Zn^2+^ concentration, and incubation time were set at pH 9, 1.49 mM, and 10 min, respectively. These optimal parameters were used for the following studies.

### 3.3. Examination of Quantitative Analysis

**Figure 5:**
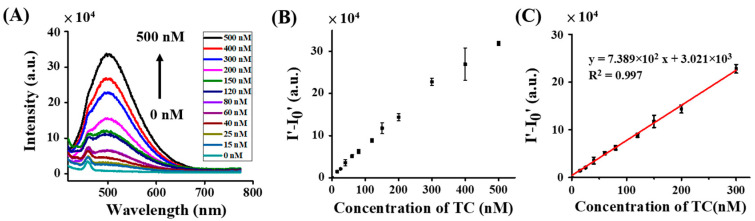
Fluorescence spectra of the samples containing (**A**) Zn^2+^ (15 µL, 31.3 mM) with the addition of different concentrations of TC (0–500 nM, 300 µL). (**B**) The corresponding calibration curve obtained by plotting the difference of the fluorescence intensity of the samples containing Zn^2+^ with (I′) and without (I_0′_) adding TC versus the concentration of TC. (**C**) Calibration plot with the linear dynamic range. I′ and I_0′_ were individually obtained from their differences of the corresponding fluorescence intensity at the wavelength of 500 nm to 750 nm in the resultant fluorescence spectra.

The feasibility of using the developed method for quantitative analysis was further investigated. Figure 5A shows the representative fluorescence spectra of the samples containing TC (0–500 nM) obtained by using Zn^2+^ as the sensing probe. The fluorescence intensity at the maximum emission wavelength of 500 nm increased as the concentration of TC in the samples increased. Figure 5B shows the corresponding calibration graph by plotting the difference of the fluorescence intensity between the samples and the blank versus the concentration of TC. The linear dynamic range was 15–300 nM (R^2^ = 0.997, y = 7.389 × 10^2^x + 3.021 × 10^3^; Figure 5C). The LOD was estimated to be ~7.0 nM, which was calculated based on 3σ/slope, in which σ indicates the standard deviation of the intercept on the Y axis. The LOD was lower than the allowed concentration established by the European Union (i.e., ~225 nM). In addition, the LOD was lower than those obtained from most existing analytical methods [43,44,45]. It was higher than that when magnetic Fe_3_O_4_@ZnS:Mn^2+^ QDs were used as sensing probes toward TC (i.e., 1.2 nM) [46]. Table S1 lists the comparison of existing fluorescence-based sensing methods against TC with our method in terms of sensing probes, methods, LODs, and analysis time. Although the LOD of our current method was not the lowest, the incubation time was relatively short. Furthermore, the preparation of the sensing probe (i.e., Zn^2+^ in Tris) was simple, without the requirement of a long synthesis time to fabricate additional ligands to chelate with Zn^2+^.

### 3.4. Examination of the Effects from Interferences

**Figure 6:**
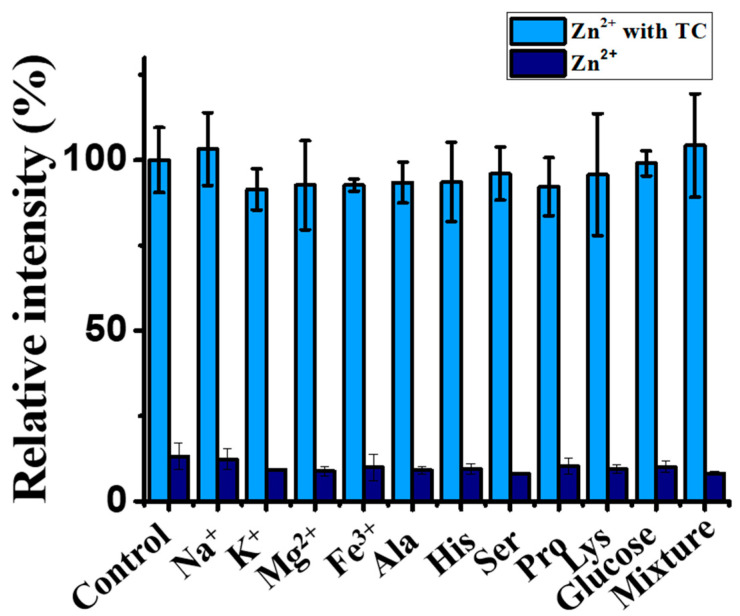
Examination of interference effects. Bar graphs obtained from the summarized results shown in Supplementary Materials Figure S4A,B.

We further examined whether the interference species that are commonly found in real samples could affect our sensing results when using our Zn^2+^-based sensing probes against TC. Metal ions (Na^+^, K^+^, Mg^2+^, and Fe^3+^) and biomolecules (alanine, histidine, serine, proline, lysine, and glucose) that are commonly found in real samples were selected as interference species. The concentration of these interference species was five-fold higher than that of TC in the samples. Supplementary Materials Figure S4A,B show the representative fluorescence spectra of the samples containing Zn^2+^ with and without the addition of TC (100 nM) in the absence and presence of interference species. Figure 6 shows the summarized results from three replicates. The presence of these interference species did not remarkably affect the sensing results of Zn^2+^ against TC.

### 3.5. Examination of the Precision and Accuracy of the Developed Method

We also investigated the precision and accuracy of the developed method. The sample containing TC (50 nM) was prepared initially. Two people used the same sample to conduct the experiments three times in the morning and three times in the afternoon for 5 days. Table 1 lists the summarized results. Accordingly, the accuracy was estimated to be 96.4%, whereas the precision was ~13.0% from 60 datasets. The results indicated that our method had acceptable accuracy and precision.

### 3.6. Analysis of Simulated Real Samples

**Figure 7:**
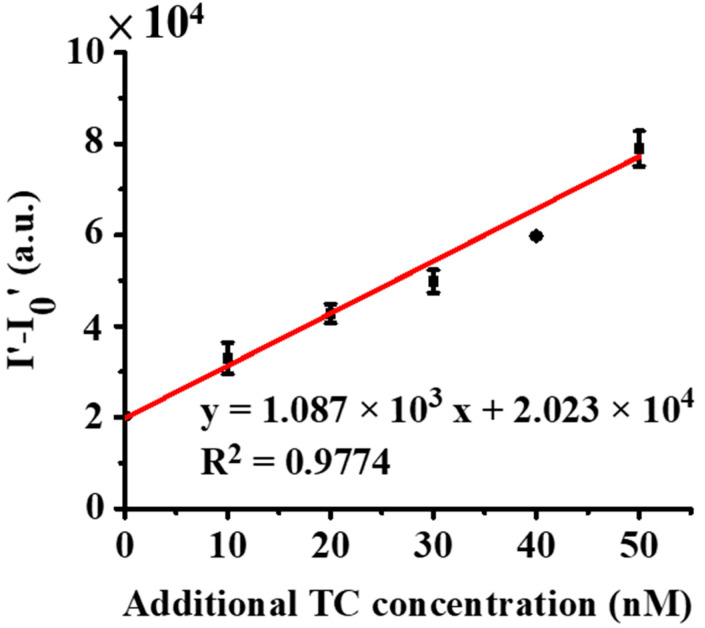
Quantitative analysis of TC (20 nM) spiked in a chicken broth sample obtained from the standard addition method. Calibration curve obtained from three replicates (Figure S5A–C) by plotting the difference of the fluorescence intensity of the samples containing Zn^2+^ with (I′) and without (I_0′_) adding TC versus the concentration of TC. I′ and I_0′_ were individually obtained from their differences of the corresponding fluorescence intensity at the wavelength of 500 nm to 750 nm in the resultant fluorescence spectra.

In demonstrating the feasibility of using the developed method for TC detection in real samples, chicken broth was selected as the model sample. TC (20 nM) was spiked to the as-prepared chicken broth. The standard addition method was used to determine TC in the as-prepared samples. Supplementary Materials Figure S5A–C show the resultant fluorescence spectra of the as-prepared chicken broth samples spiked with TC at different concentrations from three replicates. Accordingly, Figure 7 shows the resulting calibration curve (R^2^ = 0.9774, Y = 1.087 × 10^3^x + 2.023 × 10^3^) by plotting the difference in fluorescence intensity (I_500_–I_750_) between Zn^2+^ with TC and Zn^2+^ only versus the added concentration of TC. The concentration of TC was estimated to be 18.61 nM, which was only a ~6.95% difference from that of the known value. The results indicated that our method can be potentially used in the determination of TC in complex real-world samples.

## 4. Conclusions

Although existing fluorescence-based sensing methods for the detection of TC are quite sensitive, most fluorescence-sensing probes require complicated and time-consuming fabrication steps. In this study, a facile switch-on fluorescence method using Zn^2+^ as the sensing probe has been developed. Zn^2+^-based probes are prepared by simply dissolving Zn^2+^ in Tris buffer at pH 9. Apart from the short preparation time (i.e., a few minutes) for the generation of sensing probes, the sensing performance of our approach is comparable or even better than other fluorescence-sensing methods. Our method only requires 10 min of incubation time, and the entire analysis time only takes ~20 min. The LOD toward TC is as low as ~7 nM. In addition, the developed method is simple, and it has rapid response and low LOD. Given that Zn^2+^-TC conjugates can emit bright fluorescence, we believe that they can be further used as fluorescent labeling agents against bacteria. Moreover, TC is a broad-spectrum antibiotic, which can penetrate bacterial cell walls by passive diffusion [47]. Thus, the antibacterial effectiveness of Zn^2+^-TC conjugates is worthy of investigation. Efforts should be further devoted to demonstrating the possibility of these potential applications.

## Figures and Tables

**Table 1:** Evaluation of the precision and accuracy of the developed method.

		Person A					Person B				
		Day 1	Day 2	Day 3	Day 4	Day 5	Day 1	Day 2	Day 3	Day 4	Day 5
morning	1	51.0	43.0	39.1	46.1	49.3	48.2	36.8	39.1	56.3	43.7
	2	46.8	37.6	42.7	45.6	56.0	47.2	45.2	40.5	43.5	42.7
	3	43.8	55.7	42.5	47.7	49.9	48.6	39.4	39.0	56.2	35.3
afternoon	1	50.1	45.9	41.8	44.9	59.4	50.4	42.8	41.7	50.7	39.1
	2	43.1	55.3	40.5	44.0	60.4	38.5	43.9	49.3	57.4	42.8
	3	41.7	47.6	40.2	44.3	51.7	41.8	44.5	42.3	55.5	43.2
mean (*n* = 6)		46.1	47.5	41.1	45.5	54.5	45.8	42.1	42.0	53.3	41.1
SD		3.5	6.4	1.3	1.2	4.4	4.2	3.0	3.5	4.9	3.0
RSD (%)		7.7	13.5	3.1	2.7	8.1	9.2	7.2	8.3	9.1	7.4
Intermediate precision Person A (*n* = 30 for each person)			mean	46.9			Person B	mean	44.9		
			SD	5.8				SD	5.9		
			RSD (%)	12.4				RSD (%)	13.1		
Precision (*n* = 60)			mean	45.9							
			SD	6.0			Accuracy (*n* = 60)		96.4%		
			RSD (%)	13.0							

## Data Availability

The data presented in this study are available in Supplementary Materials.

## Supplementary Materials

The following supporting information can be downloaded at: https://www.mdpi.com/article/10.3390/molecules27238403/s1, Figure S1: UV–Vis absorption spectra of the samples containing TC without and withthe addition of Zn^2+^; Figure S2: Examination of pH effects.; Figure S3: Optimization of experimental parameters; Figure S4: Examination of interference effects; Figure S5: Three replicated fluorescence spectra of the as-prepared chicken broth samples containing TC; Table S1: Comparison of the developed method with the existing methods.

## Sample Availability

Samples of the compounds are not available from the authors.
