# Supplementary material for: Zinc Ion-Based Switch-on Fluorescence-Sensing Probes for the Detection of Tetracycline
Source: Molecules. 2022 Dec 1;27(23):8403. doi: 10.3390/molecules27238403 (PMC9739377; doi:10.3390/molecules27238403)
Supplement: Supplementary file 1 [file molecules-27-08403-s001.zip › molecules-2025975-supplementary.pdf]

Table S1. Comparison of the developed method with the existing methods.

**Sensing probes**	**Method**	**Linear dynamic range**	**LOD (nM)**	**Analysis time**	**Ref.**
Ir(III)@SiNPs-Eu^{3+}	Ratiometric fluorescence	0.01–20 µM	4.9 nM	Few minutes	[23]
His-AuNCs/Eu^{3+}	Ratiometric fluorescence	10 nM -60 µM	4 nM	10 min	[26]
Eu^{3+}-AgNPs	Fluorescence enhancement	0.01–10 µM	4 nM	Few minutes	[28]
AuNPs-SiO_2/Eu^{3+}	Fluorescence enhancement	0 – 6 µM	83.1 nM	Few minutes	[13]
Eu^{3+}-CQDs	Fluorescence quenching	0.5 – 200 µM	300 nM	Few minutes	[19]
g-C_3N_4/Eu^{3+}	Ratiometric fluorescence	0.25-80 µM	6.5 nM	Few minutes	[29]
Zn^{2+}coordination polymers (CPs)	Fluorescence quenching	1-60 µM	0.86 µM/1.41 µM	Few minutes	[14]
Zn^{2+} coordination polymers (CPs)	Fluorescence enhancement	0.2-6.0 µM	12 nM	5 min	[30]
Zn^{2+}-MOFs	Fluorescence quenching	1-10 µM	0.15 µM	Few minutes	[39]
Zn^{2+}-MOFs	Fluorescence quenching	0-75 µM<br>0-10 µM	0.234 µM/<br>0.377 µM	Few minutes	[40]
Zn^{2+}-MOFs	Fluorescence enhancement	0.02-13 µM	17 nM	1 min	[31]
Fe_3O_4@ZnS:Mn^{2+} QDs	Fluorescence quenching	10 – 700 nM	1.2 nM	Few minutes	[46]
Eu^{3+} functionalized ZnO QDs	Ratiometric fluorescence	5 nM–3 µM	4 nM	100 s	[15]
Zn^{2+}-Tris complex	Fluorescence enhancement	15-300 nM	~7 nM	10 min	This work

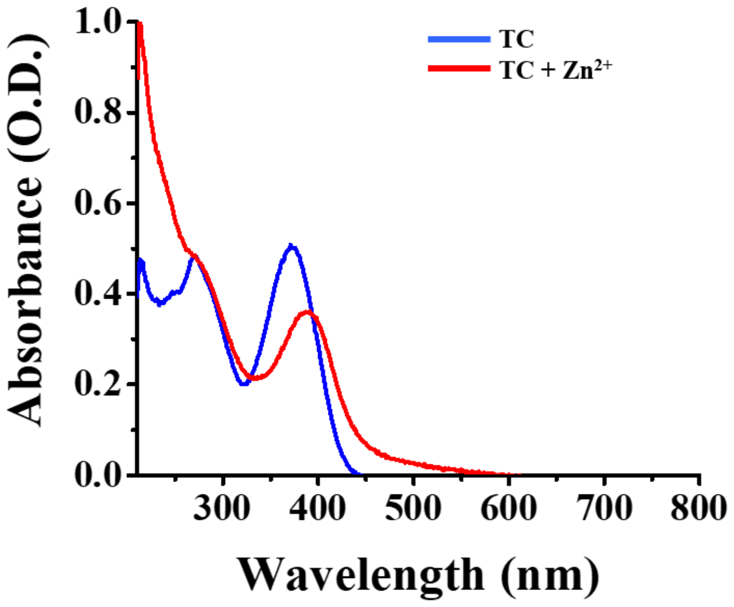

Figure S1. UV–Vis absorption spectra of the samples containing TC (40 μM) without (blue) and with (red) the addition of Zn^{2+} (1.49 mM).

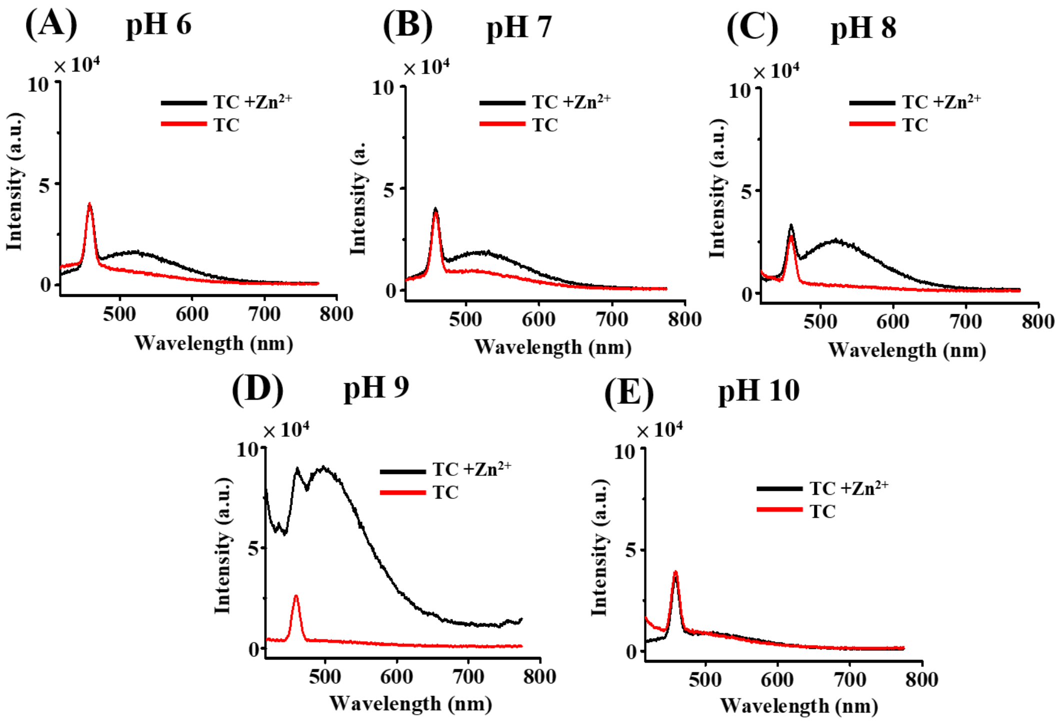

Figure S2. Examination of pH effects. (A)-(E) Representative fluorescence spectra of the samples containing TC (0.1 μM, 300 μL) obtained before (red) and after adding Zn^{2+} (1.49 mM) (black) at different pH conditions for min followed by examination of fluorescence spectroscopy (λ_{ex}= 397 nm).

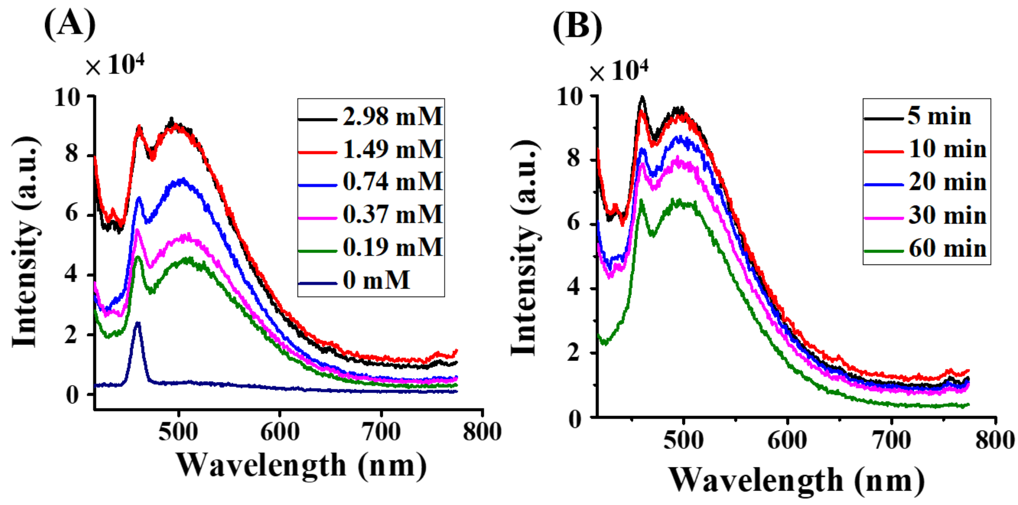

Figure S3. Optimization of experimental parameters. (A) Representative fluorescence spectra of the samples containing TC (0.1 μM, 300 μL) obtained before and after adding Zn^{2+} at different concentrations (0.19-2.98 mM) for 10 min (λ_{ex}= 397 nm). (B) Representative fluorescence spectra of the samples containing TC (0.1 μM, 300 μL) obtained after adding Zn^{2+} (1.49 mM) for different incubation times (λ_{ex}= 397 nm).

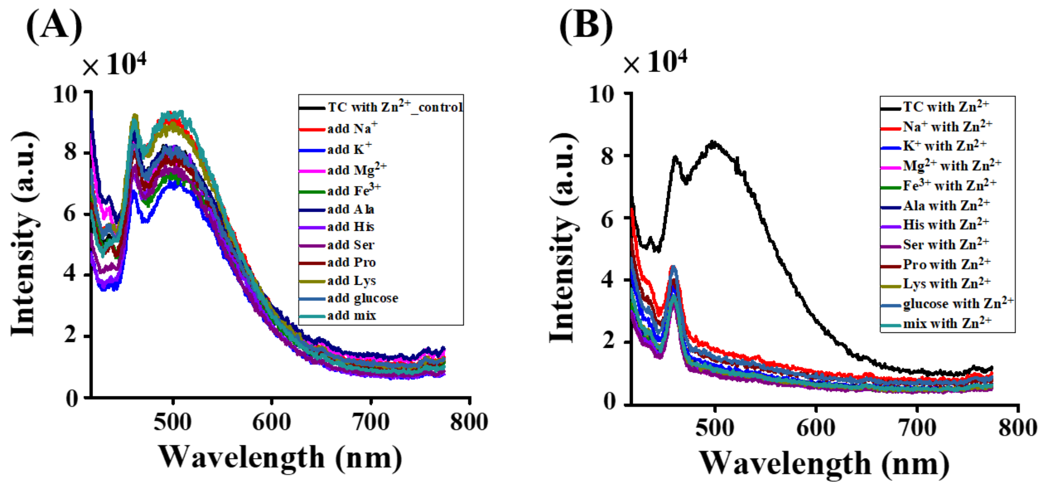

Figure S4. Examination of interference effects. Fluorescence spectra of the samples containing (A) TC (300 μL, 0.1 μM) with adding Zn^{2+} (1.49 mM) in the absence (black) and in the presence of interference species (15 μL, 10 μM) (λ_{ex}= 397 nm). (B) Fluorescence spectra of the samples containing Zn^{2+} (1.49 mM) with the presence of TC (0.1 μM) (black) and other interference species (0.5 μM) (λ_{ex}= 397 nm).

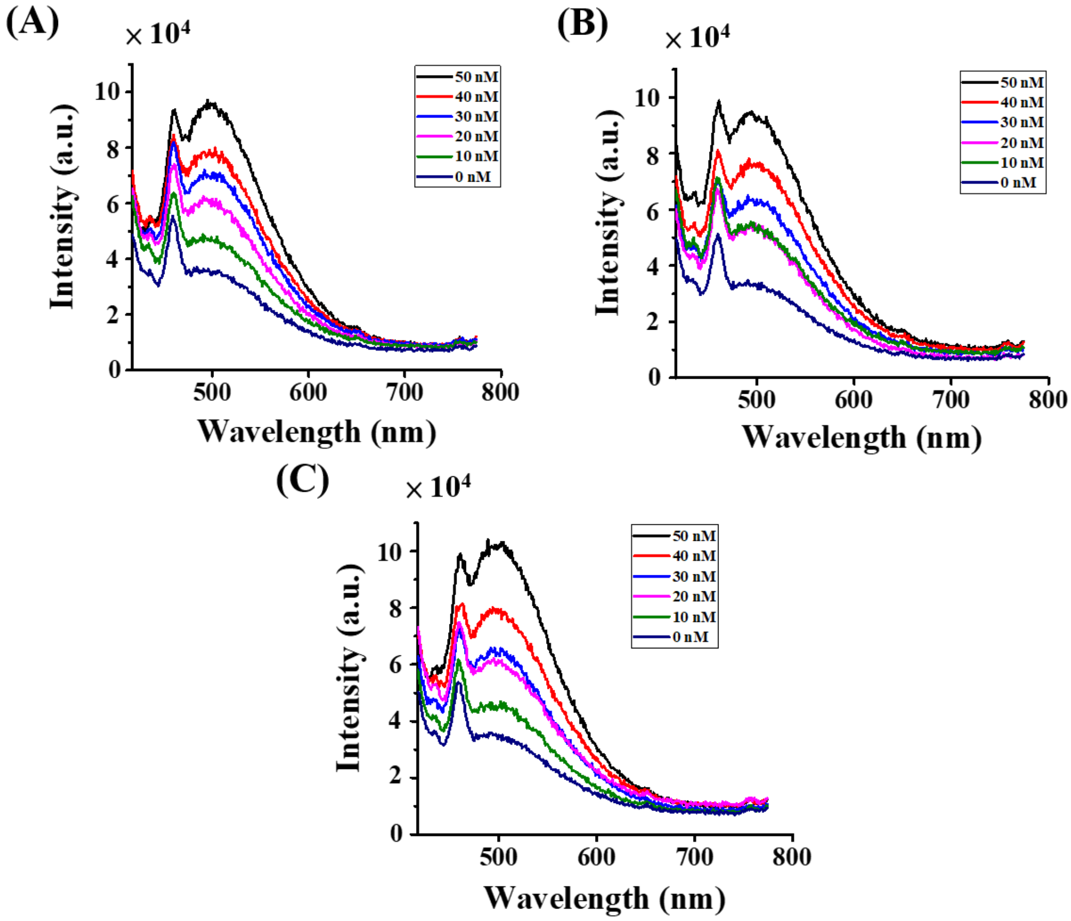

Figure S5. (A)-(C) Three replicated fluorescence spectra of the as-prepared chicken broth samples containing TC (20 nM) obtained by the standard addition method with the addition of TC at different concentrations (0-50 nM) (λ_{ex}= 397 nm).
